# Breaking the cycle: Preventing workplace violence against healthcare workers

**DOI:** 10.1002/jhm.70167

**Published:** 2025-09-21

**Authors:** Christina T. Vo, Karen Brooks, Emmanuel King

**Affiliations:** ^1^ Department of Patient Safety Hospital of the University of Pennsylvania Philadelphia Pennsylvania USA; ^2^ Department of Medical Transplant—Advanced Lung Disease University of Pennsylvania Philadelphia Pennsylvania USA; ^3^ Department of Nursing Hospital of the University of Pennsylvania Philadelphia Pennsylvania USA; ^4^ Perelman School of Medicine at the University of Pennsylvania Philadelphia Pennsylvania USA; ^5^ Department of Medicine, Division of Hospital Medicine University of Pennsylvania Philadelphia Pennsylvania USA

## Abstract

Workplace violence (WPV) in healthcare is a growing crisis, and hospital‐based approaches are urgently needed. We review two recently published efforts and describe our own pathway to address WPV. Sahota et al. implemented a specialized team and leveraged individualized care plans for high‐risk patients, while Littlefield et al. took a policy‐driven approach, enabling administrative discharge of disruptive patients. Our pathway utilized patient‐centered anticipatory guidance, staff de‐escalation training, and a behavioral emergency response. All three approaches illustrate that WPV prevention must move beyond reactive protocols and pivot to proactive, patient‐informed solutions.

*‐A patient with anxiety/depression and substance use disorder is admitted to the medicine service for cellulitis. A female resident enters his room and notices he is fidgeting and that his speech is pressured. He suddenly grabs her, throws her on the bed, and attempts to sexually assault her*.

*‐A surgical ICU nurse is changing a chest tube dressing when, without warning, he is suddenly punched in the face*.

Workplace violence (WPV) is defined as “…verbal or written threats; physical or verbal harassment; direct physical assaults; and homicide.”^1^ Healthcare workers are at substantial risk of WPV, accounting for 50% of incidents.^2^ Centers for disease control (CDC) vital signs reported that violence against healthcare workers doubled between 2018 and 2022, from 6% to 13%.^3^ While guidance from the Joint Commission and Centers for Medicare and Medicaid Services are important resources, a more systematic approach is needed. In this *Journal*, two innovative studies highlight very different approaches to WPV prevention.

Patients with behavioral dysregulation and underlying mental health disorders have a higher propensity for violence toward healthcare workers that may be best addressed by comprehensive inpatient behavioral health teams.^4^ While Sahota et al. note that at their institution, training and support are available to manage violent or disruptive events, they lacked a process for prevention.^5^ The investigators focused on four patients who were involved in a disproportionate number of past events (67 violent behavior escalation events in the prior year). A therapeutic violence mitigation (TVM) core clinician team, comprised of a psychiatrist, hospitalist, psychologist, and a team of behavioral clinical specialist (BCS) nurses, created individualized care plans for these high‐risk patients and filed them in the electronic medical record (EMR). When this team was alerted that one of the pilot patients was admitted, they facilitated implementation of the plan by nursing staff and providers. After 6 months, the WPV events for the four patients plummeted from the baseline of 67 down to just two events.

Due to the impressive early success of Sahota's program, they rapidly expanded to 100 patients and created a standardized workflow that could be used by all staff and providers. While we commend this study, we wonder: Did the Hawthorne effect skew the early results? Will replication of this study at other healthcare institutions be difficult due to the funding and specialized personnel needed for a TVM team? Furthermore, the alerts that informed the TVM team appeared to utilize behavioral flag alerts in the EMR. Recent studies raise concern for the potential of inequitable care when behavioral flags are present in the charts of Black patients and those from lower socioeconomic backgrounds.^6^, ^7^ Despite these questions, this is important work we hope to see emulated.

Littlefield et al. implemented a very different, staff‐centered, policy‐driven intervention.^8^ A group of stakeholders reviewed the current state of WPV event management, evaluated gaps in the process, created a driver diagram, and developed a pathway for “administrative discharge.” Over 12 months, five patients were deemed appropriate for administrative discharge. However, despite the interdisciplinary and rigorous nature of their design process, notably absent as a stakeholder was the patient, who stands to be the most affected by an administrative discharge. By employing a policy that primarily protects the institution, the patient's perspective has the potential to be marginalized, which eliminates the opportunity to explore and identify the root of “disruptive” behaviors. People often resort to maladaptive behaviors when they perceive a grievance or that they are being treated unfairly. An intervention that may benefit the institution more than the patient may inhibit effective collaboration and thereby may eliminate the curiosity necessary to identify mutual interests and values.^9^

A review of the patients in Littlefield's administrative discharge pilot revealed a striking pattern: four of five patients had substance use disorder (SUD). This may provide a starting point for future iterations of this study and underscores the need to consider “disruptive behaviors” as a symptom of unmet needs. A policy‐driven model that does not account for the behavioral and clinical complexities of SUD risks reinforcing stigma, and misses opportunities for therapeutic engagement. Tailored interventions—such as specialized de‐escalation training, integrated addiction or harm reduction services, and trauma‐informed care—are essential to both protect staff and to support vulnerable patients in crisis and may lead to decreased reliance on administrative discharge.

We are grateful to also have the support of our hospital's executive leadership to develop a multidisciplinary approach that balances the needs of the patient with the safety of the staff. Alarmed by a steady increase in WPV events, particularly on general medicine/hospitalist units, we developed a WPV prevention clinical pathway centered around anticipatory guidance.^10^ Anticipatory guidance is a proactive approach often seen in nonmedical settings like college loan counseling and retirement planning; in healthcare, it may be utilized for education during prenatal care or before procedures. Our novel approach was a patient handout developed with input from our Patient/Family Caregiver Center and provided with the standard patient admission packet. The handout validates the stressfulness of hospitalization, specifically referencing emotions that patients may experience and utilizing pictures akin to the Wong–Baker FACES pain scale to depict anxiety, sadness, confusion, loneliness, and feeling overwhelmed. Highlighted are readily available stress management resources (a visit from pastoral care or therapy dogs, a Reiki session, coloring books, puzzles, reading material, etc.). In our experience, utilizing anticipatory guidance to better acknowledge the span of emotions a patient may experience during hospitalization is a unique foundational step toward mitigating violent or disruptive behavior. A 2‐month pilot on four general medicine units reduced reported WPV events from an incidence rate of 3.017 WPV events/1000 patient beds to 1.823 WPV safety events/1000 patient beds. Just as important as the promising early data was the positive feedback from staff who felt that proactively acknowledging the stress of hospitalization and giving patients tools led to meaningful conversations and gratifying opportunities for early de‐escalation.

We have since expanded our pathway, utilizing a three‐tiered public health prevention framework that extends from the primary prevention/anticipatory guidance above to secondary prevention (de‐escalation techniques using Crisis Prevention Institute [CPI] training and CDC best practices) and tertiary prevention (behavioral emergency response [BER]). BER may be activated when secondary prevention has been unsuccessful or at any time providers or staff feel unsafe. Our response algorithm was developed by a team of nurses, providers, security, psychiatry, and pharmacy who mapped out a pathway to reduce delays in response to WPV events, which were felt to be strong contributors to in‐the‐moment chaos and even postevent stress. Our current work focuses on streamlining BER activation, timely mobilization of team members, assuring restraints arrive rapidly with security staff, and optimizing timely medication access. During a 1‐year pilot of our pathway on 10 units across medicine and neuroscience, WPV incidents on the medicine units decreased by 48%. While the neuroscience units had an initial reduction in WPV events, these events later increased, which we are investigating to pinpoint the root cause. We are now working on scaling this WPV prevention pathway across all units in our 1000‐bed academic center.

We applaud the work of our colleagues and are excited to be part of the WPV prevention movement. The remarkable early success of Sahota's intervention highlights the positive changes that can occur with a patient‐centered approach via individualized care plans. We would love to see a longitudinal study of its impact on an expanded patient population and evaluation for the potential for unintended implicit bias due to the use of EMR flags. Littlefield's protocol for administrative discharge has elements that many hospitals may want to consider adopting to support providers, but we caution against overlooking the unmet needs of patients in the process. We have plans to expand our WPV initiatives, including the expansion of our BER, simulation training, and evidence‐based medication management algorithms. As the incidence of WPV rises, hospitalists and their interdisciplinary partners must act with urgency to implement thoughtful, evidence‐based interventions that protect healthcare workers while preserving dignity and compassion in patient care. The path forward lies in balancing safety with empathy—and in recognizing that true prevention requires curiosity, collaboration, and systemic change.

## CONFLICT OF INTEREST STATEMENT

The authors declare no conflicts of interest.
